# Should high-flow through nasal cannula be used during bronchoscopy in critically ill patients with hypoxemic acute respiratory failure?

**DOI:** 10.1186/s44158-021-00001-y

**Published:** 2021-09-04

**Authors:** Federico Longhini, Andrea Bruni, Giuseppe Saraco, Eugenio Garofalo, Giorgio Conti

**Affiliations:** 1 Anesthesia and Intensive Care, “Mater Domini” University Hospital, Department of Medical and Surgical Sciences, “Magna Graecia” University, Viale Europa, 88100 Catanzaro, Italy; 2 Department of Emergency Medicine, Anaesthesia and Intensive Care, University Hospital Agostino Gemelli IRCCS, Rome, Italy

**Keywords:** High-flow nasal cannula, Bronchoscopy, Intensive care unit, Acute respiratory failure

## Abstract

Flexible fiberoptic bronchoscopy (FOB) is an invasive procedure with diagnostic and/or therapeutic purposes commonly used in critically ill patients. FOB may be complicated by desaturation, onset or worsening of the respiratory failure, and hemodynamic instability due to cardio-respiratory alterations occurring during the procedure. Increasing evidences suggest the use of high-flow through nasal cannula (HFNC) over conventional oxygen therapy (COT) in critically ill patients with acute respiratory failure (ARF). Indeed, HFNC has a rationale and possible physiologic advantages, even during FOB. However, to date, evidences in favor of HFNC over COT or continuous positive airway pressure (CPAP) or non-invasive ventilation (NIV) during FOB are still weak. Nonetheless, in critically ill patients with hypoxemic ARF, the choice of the oxygenation strategy during a FOB is challenging. Based on a review of the literature, HFNC may be preferred over COT in patients with mild to moderate hypoxemic ARF, without cardiac failure or hemodynamic instability. On the opposite, in critically ill patients with more severe hypoxemic ARF or in the presence of cardiac failure or hemodynamic instability, CPAP or NIV, applied with specifically designed interfaces, may be preferred over HFNC.

## Background

Flexible fiberoptic bronchoscopy (FOB) is an invasive procedure with diagnostic and/or therapeutic purposes, used since a long time in patients with airway or lung parenchyma disorders of varying etiology and severity. In critically ill patients, FOB is commonly performed to remove plugs of secretions occluding the airway in the presence of abundant secretions or ineffective cough, or in association with the bronchoalveolar lavage (BAL) to diagnose a vast array of lung diseases [1].

Although considered safe, FOB may be characterized by the occurrence of adverse events related to the maneuver, such as desaturation, onset or worsening of the respiratory failure, and hemodynamic instability. In critically ill patients, FOB with BAL may deteriorate gas exchange and the arterial partial pressure of oxygen (PaO_2_) can drop of 10–20 mmHg [1, 2]. Noteworthy, after FOB with BAL, up to 32% of non-intubated patients may experience a clinical adverse event requiring an escalation of the ventilatory support or even intubation [3].

## Cardio-respiratory alterations during FOB

When performing FOB (with or without BAL), the clinician should be aware of some occurring alterations of respiratory mechanics and hemodynamic status (Table 1).

**Table 1:** Cardio-respiratory effects of flexible bronchoscopy

Respiratory system	Hemodynamic status
• Increase of airway resistances • Work of breathing enhancement • Alveolar de-recruitment and lung collapse (in particular during suctioning and BAL) • Worsening of gas exchange	• Alterations of intrathoracic pressures • Increased sympathetic stimulation • Cardiac distress (in particular in cardiopathic, fragile, and unstable patients)

*BAL*, bronchoalveolar lavage

First, the fiberscope occupies approximately the 10% of the cross-sectional area of the trachea and the 15% at the cricoid ring. As a consequence, the fiberscope acts as a foreign body, increases both the inspiratory and expiratory airway resistances, enhances the work of breathing, and may induce dynamic hyperinflation with an augmented functional residual capacity [4, 5]. In addition, when suctioning is applied, the airway and alveolar pressures drop to zero, or even negative. In this event, suctioning induces a loss of end-expiratory lung volume, alveolar de-recruitment, and atelectasis, resulting in increased shunt and venous admixture and, finally, worsening the gas exchange [6]. Such respiratory changes fully revert after FOB in a period of time up to several hours, in the most severe patients [4, 6].

These modifications are even more prominent in the case of FOB with BAL. In fact, when BAL is performed, some aliquots of sterile saline solution are injected and then gradually aspirated back into the syringe through the internal channel of the fiberscope. Commonly, the injected volume is not completely recovered back, and a large part remains in the alveola. Therefore, the end-expiratory volume of the portion of parenchyma involved in the maneuver is reduced well below the functional residual capacity [4, 7], leading to alveolar collapse and ventilation-perfusion mismatch [8, 9].

Furthermore, FOB may alter the hemodynamic status because of the complex interplay that exists between respiratory and cardiovascular systems. It is well known that the application of positive intrathoracic pressure reduces the stroke volume by increasing the right ventricular afterload and, to some extent, by reducing the preload. The interplay is more and more complex in patients with spontaneous breathing activity, whose respiratory efforts affect intrathoracic pressure and venous return to the right ventricle [10]. The insertion of the fiberscope potentially alters the hemodynamic status through changes of the intrathoracic pressure secondary to dynamic hyperinflation or, on the opposite, airway suctioning and augmented respiratory effort. To further complicate the circumstances, sympathetic stimulation during FOB is also high. As a result, the cardiac output increases by 50% and it returns to its baseline in 15 min after completion of the procedure [4, 11, 12]. The variation of the hemodynamic status is of particular importance in unstable, fragile, or cardiopathic patients. In fact, it has been reported that FOB may cause a dangerous cardiopulmonary distress, associated with electrocardiographic alteration, in up to 21% of awake patients [12].

## High-flow through nasal cannula and its rationale during FOB

In the last decade, high-flow through nasal cannula (HFNC) has been increasingly used over conventional oxygen therapy (COT) in daily clinical practice. HFNC consists of administration of elevated flows (up to 60 L/min) of air/oxygen admixtures, heated (at temperatures ranging from 31 to 37 °C) and fully humidified (up to 44 mg H_2_O/L), providing an inspired oxygen fraction ranging from 21 to 100% [13].

The use of HFNC has a rationale and possible physiologic advantages in spontaneous breathing critically ill patients. First, HFNC determines a washout effect from carbon dioxide (CO_2_) of the pharyngeal dead space, which is proportional to the flow applied. In particular, every increment of 1 L/min of the flow applied through HFNC determined a 1.8-mL/s increase of the clearance in the nasal cavities. Furthermore, the washout effect is also time-dependent: the lower is the respiratory rate (and therefore the longer the expiratory time), the higher is the washout effect [14]. This mechanism of action translates into a reduced work of breathing, when compared to COT [15, 16].

Second, HFNC generates a small amount of positive pharyngeal airway pressure during expiration depending on the flow rate, the upper airway anatomy, the breathing through the nose or mouth, and the size of the cannula in relation to the nostrils [13]. This low expiratory pressure translates into a small alveolar distending pressure that improves the end-expiratory lung volume and oxygenation in critically ill patients with different conditions of acute respiratory failure (ARF) [17–20].

Third, HFNC guarantees a more stable inspired oxygen fraction (FiO_2_), as compared to COT through nasal prongs or masks. When the patient’s inspiratory peak flow increases to an extent that exceeds the flow delivered by COT systems, FiO_2_ is no more predictable. On the opposite, HFNC guarantees the set FiO_2_ in every patient with an inspiratory peak flow up to 60 L/min (i.e., the maximal flow delivered by the HFNC system) [21].

Finally, the HFNC decreases the resistive breathing effort, reducing the upper airway resistance [13].

## The use of HFNC during FOB

Based on the aforementioned mechanisms, HFNC could play a beneficial role in the prevention or reduction of cardio-respiratory alterations induced by FOB and, at the end, may diminish the occurrence of some adverse events.

HFNC guarantees an acceptable oxygenation during FOB with BAL in critically ill patients with moderate [22] to more severe [23] hypoxemic ARF of varying etiology. In one observational study, relevant (< 88%) desaturations occurred in two out of 30 patients [23], whereas in another one no patients required the interruption of the procedure for desaturations [22]. Noteworthy, in the former study, two patients underwent endotracheal intubation and mechanical ventilation because of worsening of the respiratory disease and gas exchange within 24 h after FOB with BAL [23], whereas in the latter, one out of five patients required the application of continuous positive airway pressure (CPAP) 16 h after the procedure until 5 days later [22]. HFNC was also judged to be comfortable during the procedure [23] and with a similar occurrence of hemodynamic impairment to COT [24]. Noteworthy, HFNC is superior to COT during FOB only when the delivered flow is set at 60 L/min [8, 25].

Long before the advent of HFNC, CPAP or non-invasive ventilation (NIV) was commonly applied in high-risk hypoxemic patients during FOB [2, 26, 27]. In 40 patients with hypoxemic ARF of moderate severity, NIV through face mask improved, as compared to baseline, the oxygenation from 15 min after its application, throughout the entire bronchoscopy, and till 50 min after the procedure. On the opposite, HFNC kept the oxygenation unmodified compared to its baseline [28]. Of note, one patient out of 20 randomized to receive HFNC required intubation soon after the end of the procedure for severe gas exchange deterioration, whereas three out of 20 patients randomized to NIV required intubation within 24 h after FOB [28].

Another recent trial randomized 51 patients with less severe hypoxemic ARF to receive HFNC or NIV during FOB [29]. HFNC and NIV were both well tolerated and effective to guarantee oxygenation. However, compared to HFNC, NIV provided more stable oxygenation and hemodynamics during and after the procedure in patients with a PaO_2_ < 60 mmHg on room air [29].

## Possible drawbacks during clinical practice

**Fig. 1:**
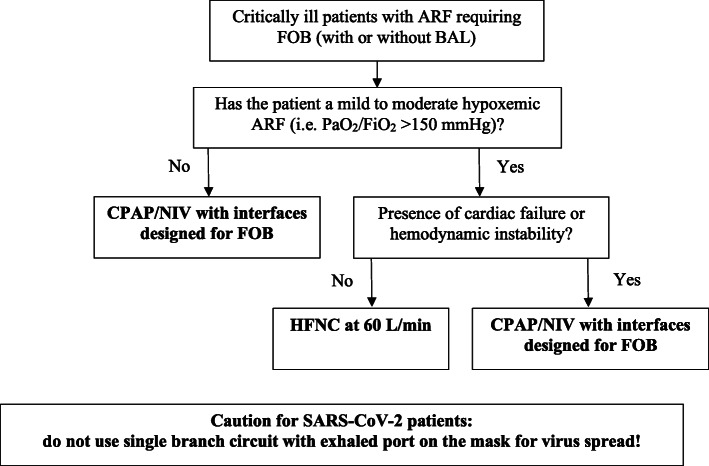
Possible approach for FOB in ICU in non-intubated patients. ARF, acute respiratory failure; FOB, flexible fiberoptic bronchoscopy; BAL, bronchoalveolar lavage; PaO_2_/FiO_2_ ratio between arterial partial pressure of oxygen and inspired oxygen fraction; CPAP, continuous positive airway pressure; NIV, non-invasive ventilation; HFNC, high-flow oxygen through nasal cannula; SARS-CoV-2, severe acute respiratory syndrome coronavirus 2

In critically ill patients with hypoxemic ARF, the choice of the oxygenation strategy is challenging. To date, evidences in favor of HFNC or NIV lack. When choosing a device, the physician should consider several aspects. First, the use of one strategy, rather than another, may interfere with the access of the FOB. For example, HFNC limits the possibility to use the nasal route because of the presence of large bore nasal prongs. Furthermore, the positive expiratory airway pressure generated by HFNC would be significantly reduced during open mouth breathing [30]. Recently, it has been shown that in outpatients undergoing FOB with BAL, HFNC prevents oxygenation worsening by avoiding end-expiratory loss of lung volume and preserves the same tidal volume with a lower diaphragm activation, even if the bronchoscope was introduced through the mouth [25]. On the opposite, the use of NIV may be problematic due to the availability of interfaces with dedicated ports for the insertion of the bronchoscope. In addition, NIV may be also affected by poor patient-ventilator synchrony during FOB, worsening the comfort to the patient [31]. Based on the current, though limited, literature, we propose a possible approach to FOB (with or without BAL) for non-intubated critically ill patients with ARF (Fig. 1).

Second, as mentioned above, FOB induces cardiovascular alterations, which may precipitate fragile heart conditions. At this regard, CPAP and NIV provide a positive airway pressure through the entire respiratory cycle, while HFNC does not [30]. Cardiovascular benefits of positive intrathoracic pressure are well known, and, for example, CPAP/NIV are strongly recommended in patients with cardiogenic pulmonary edema [32]. On the opposite, this is not guaranteed by HFNC. These features may suggest the use of CPAP or NIV over HFNC as more appropriate oxygenation strategies in patients with concomitant cardiac diseases and/or heart failure (Fig. 1).

Finally, during the SARS-CoV-2 pandemic, the spread of the virus during a high-risk procedure, such as FOB, should also be considered in the choice of the interface. If the mask well-fits to the patient and air leaks are limited, the exhaled air dispersion is similar to that reported during HFNC treatment and it has been reported in a range between 172 and 332 mm [33]. Similarly, during helmet NIV, the exhaled air leaks through the neck-helmet interface with a radial distance of 150 to 230 mm [34]. Caution must be posed when the patient is receiving NIV through a mask with intentional leaks through the exhalation port and single-branch circuit: in fact, the exhaled air jet could reach a distance of 916 mm [34].

## Conclusions

Although both HFNC and NIV are suggested over COT, there are no strong evidences in favor of HFNC or NIV in patients with hypoxemic ARF. While HFNC could be used in less severe cases, NIV should be preferred over HFNC in more severe patients or in the presence of cardiovascular comorbidities. Further studies are advisable to strengthen possible future indications of treatment.

## Data Availability

Not applicable

## Abbreviations

ARF: Acute respiratory failure
BAL: Bronchoalveolar lavage
CO_2_: Carbon dioxide
COT: Conventional oxygen therapy
CPAP: Continuous positive airway pressure
FiO_2_: Inspired oxygen fraction
FOB: Flexible fiberoptic bronchoscopy
HFNC: High-flow through nasal cannula
NIV: Non-invasive ventilation
PaO_2_: Arterial partial pressure of oxygen
